# Reference Intervals for Bone Impact Microindentation in Healthy Adults: A Multi-Centre International Study

**DOI:** 10.1007/s00223-022-01047-y

**Published:** 2023-02-02

**Authors:** Pamela Rufus-Membere, Kara L. Holloway-Kew, Adolfo Diez-Perez, Natasha M. Appelman-Dijkstra, Mary L. Bouxsein, Erik F. Eriksen, Joshua N. Farr, Sundeep Khosla, Mark A. Kotowicz, Xavier Nogues, Mishaela Rubin, Julie A. Pasco

**Affiliations:** 1 IMPACT- Institute for Mental and Physical Health and Clinical Translation, School of Medicine, Deakin University, Geelong, Australia; 2 Department of Internal Medicine, Hospital del Mar-IMIM, Autonomous University of Barcelona and CIBERFES, Instituto Carlos III, Barcelona, Spain; 3 Department of Internal Medicine: Division of Endocrinology and Center for Bone Quality, Leiden University Medical Center, Leiden, the Netherlands; 4 Center for Advanced Orthopedic Studies, Beth Israel Deaconess Medical Center and Department of Orthopedic Surgery, Harvard Medical School, Boston, MA USA; 5 Spesialistsenteret Pilestredet Park and Faculty of Odontology, University of Oslo, Oslo, Norway; 6 Kogod Center On Aging and Division of Endocrinology, Mayo Clinic, Rochester, MN USA; 7 Barwon Health, Geelong, Australia; 8 Department of Medicine-Western Health, The University of Melbourne, St. Albans, Australia; 9 Department of Internal Medicine, Hospital del Mar-IMIM, Pompeu Fabra University Barcelona- and CIBERFES, Instituto Carlos III, Barcelona, Spain; 10 Department of Medicine, Columbia University Vagelos College of Physicians & Surgeons, New York, NY USA; 11 Department of Epidemiology and Preventive Medicine, Monash University, Melbourne, Australia; 12 IMPACT Institute, School of Medicine, Deakin University, Geelong, VIC Australia

**Keywords:** Impact microindentation, Reference interval, Osteoporosis, Bone material strength index, Normative data

## Abstract

Impact microindentation (IMI) is a novel technique for assessing bone material strength index (BMSi) in vivo, by measuring the depth of a micron-sized, spherical tip into cortical bone that is then indexed to the depth of the tip into a reference material. The aim of this study was to define the reference intervals for men and women by evaluating healthy adults from the United States of America, Europe and Australia. Participants included community-based volunteers and participants drawn from clinical and population-based studies. BMSi was measured on the tibial diaphysis using an OsteoProbe in 479 healthy adults (197 male and 282 female, ages 25 to 98 years) across seven research centres, between 2011 and 2018. Associations between BMSi, age, sex and areal bone mineral density (BMD) were examined following an a posteriori method. Unitless BMSi values ranged from 48 to 101. The mean (± standard deviation) BMSi for men was 84.4 ± 6.9 and for women, 79.0 ± 9.1. Healthy reference intervals for BMSi were identified as 71.0 to 97.9 for men and 59.8 to 95.2 for women. This study provides healthy reference data that can be used to calculate T- and Z-scores for BMSi and assist in determining the utility of BMSi in fracture prediction. These data will be useful for positioning individuals within the population and for identifying those with BMSi at the extremes of the population.

## Introduction

Fracture resistance of bone is a function of bone mass, geometry, microarchitecture and material properties. Various diseases impact bone strength through alterations in these bone characteristics. Osteoporosis is the most common skeletal disorder, with osteoporosis-related fractures set to escalate as the global population ages. The many individuals who sustain fractures experience additional complications including ill health, disability, a reduced quality of life, and possibly even death [1–3]. The burden of bone disease is enormous, with global estimates of 158 million people aged 50 years and older at high risk for osteoporotic fracture in 2010, an estimate which is set to double by 2040 [4]. Hip fractures are by far the most devastating type of fracture, accounting for about 300,000 hospitalisations each year in the United States of America (USA) alone [5]. Data from a global systematic review conducted in 2017, estimated that health and social care costs for each fragility hip fracture in the year following fracture was USD 43,669, exceeding the estimates for acute coronary syndrome and ischaemic stroke [6].

Targeting of effective interventions depends on the ability to discriminate fracture risk. Currently, fracture risk estimates are based on assessment of bone mineral density (BMD) using radiographic imaging (dual energy X-ray absorptiometry, DXA; or quantitative computed tomography, QCT), finite element analysis based on QCT [7, 8] and magnetic resonance imaging (MRI) scans [9], and use of absolute fracture risk calculations that combine such measures with other clinical risk factors [10]. These tools are predicated on the assumption that BMD (i.e. bone quantity) and age are the dominant factors in determining bone health. The utility of other technologies such as advanced imaging (e.g. peripheral quantitative computed tomography (pQCT)/high resolution pQCT (HR-pQCT, magnetic resonance imaging (MRI)), for prediction of fracture risk remains under investigation.

Bone quantity and bone quality are considered the primary contributors to bone strength [11]. Bone quantity, synonymously referred to as bone mass, can be clinically evaluated using DXA with an output of areal bone mineral density (aBMD). Measurement of aBMD has remained the medical community’s front-line surrogate of bone strength for decades, due to the observation that fracture risk increases as aBMD decreases. However, the largest absolute number of fractures in patients do not occur in those with osteoporosis on bone density criteria [12]. Data from Australia indicate that 26.9% of women with low trauma fractures have aBMD in the normal range (T-score > − 1) [12], indicating that skeletal fragility may arise from structural or material properties of bone that are not detected by densitometry. Recently, clinical research as well as governing bodies, including the National Institute of Arthritis and Musculoskeletal and Skin Diseases (NIAMS) have indicated the need for new, different, tools to clinically assess bone health [13].

A promising new measurement method, bone impact microindentation (IMI), utilises a novel handheld device, the OsteoProbe, to assess fracture resistance of cortical bone in vivo in a minimally invasive way [14]. The potential clinical significance of the new IMI technology has been previously reported [15–21]. During IMI, bone’s resistance to a microindentation is quantified as the inverse of the indentation depth. The device quantifies the microindentation distance in bone relative to a microindentation distance into a controlled reference material and expresses the resulting ratio as the (unitless) Bone Material Strength index (BMSi). It is believed that greater indentation depth reflects less resistance to propagation of microcracks. In non-clinical testing on traditional plastic materials, BMSi was significantly correlated to both Rockwell and Vickers Hardness [22]. Previous studies have evaluated BMSi in relation to fragility fractures [23–26], chronic kidney disease [20], type 2 diabetes mellitus (T2DM) [15–17, 21], hyperparathyroidism [27], acromegaly [28], Paget’s disease [29], therapy with bisphosphonates [30] and glucocorticoid induced osteoporosis [31]. In most of these studies, BMSi was lower in the presence of disease. However, most have compared individuals with diseases that predispose to fracture, or those who have suffered a fracture, with controls who have been selected on the basis of being free of exposures that affect bone and calcium metabolism. Furthermore, since bone fragility is typically diagnosed after a fracture and most fractures occur in patients with BMD in the osteopenic range [12, 32], it is likely that the definition of healthy used in such studies may include individuals whose bone fragility remains undetected [33].

To our knowledge, no studies to date have reported reference data for BMSi in a large, heterogeneous broad-based population. The primary aim of this study was to develop reference data for BMSi in a healthy sample of men and women in the USA, Europe and Australia.

## Participants and Methods

### Study Participants

Participants for this study were healthy men and women drawn from study groups in the USA, Europe and Australia. The term “healthy” as used in this manuscript, refers to a population without comorbidities that are suspected to affect bone and are fracture-free at the time of assessment. Table 1 shows the number of participants included from each of these study centres. For all centres, participants with active disease or illness that affects bone material quality, any history of fragility fracture, or allergy to lidocaine were excluded. Each patient that was measured had their medical records reviewed by physician or trained research personnel to verify their disease state and history of fragility fracture. Each qualified clinical site received the protocol questionnaire and reviewed their measurement population according to the specific inclusion and exclusion criteria for the healthy reference interval analysis. Only eligible participants’ data were included in the analysis.

**Table 1:** Detailed list of recruitment inclusion, center details, recruitment period, number, and race/ethnicity of participants

Site ID (Region):	Healthy recruitment inclusion	Centre(s):	Recruitment period	*N*	Race/ethnicity	
					Men	Women
MAYO (USA)	Female adult (18 +), agreed to the measurement via informed consent, visiting the Clinical Research Unit (CRU) at the Mayo Clinic (Rochester, MN, USA)	Random sample of Olmsted County, MN, USA, residents, augmented by newspaper and website advertisements. Clinical Research Unit (CRU) at the Mayo Clinic (Rochester, MN, USA)	2010–2018	30	–	Caucasian, Non-Hispanic (100%)
LUMC (The Netherlands)	Male or Female adult (18 +), agreed to the measurement via informed consent, visiting the outpatient clinic of the Center for Bone Quality or the regional Fracture Liason Service (FLS) of the Leiden University Medical Center	The outpatient clinic of the Center for Bone Quality or the regional Fracture Liaison Service (FLS) of the Leiden University Medical Center. This center serves all patients of all ages that are referred or come in on their own	2013–2018	51	Caucasian (100%)	Caucasian (100%)
COLUMBIA (USA)	Female adult (18 +) visiting the Columbia University Medical Center that, is able to lie on bed, signs informed consent, has no allergy to lidocaine, and is able to be measured on the left or right tibia	Columbia University Medical Center recruited postmenopausal women through advertisement flyers. This center admits patients of all ages and disease states that are referred to them	2014–2018	22	–	Caucasian (100%), Hispanic (30%)
OSLO (Norway)	Healthy male and female adult (18 years +) controls are recruited from the Department of Endocrinology, Oslo University Hospital, having been admitted and found to have a normal skeleton. To participate as healthy controls, they must have no active illness or disease that effects bone, have no history of fragility fracture, and consent to the measurement	Department of Endocrinology, Oslo University Hospital by advertisement, self-referral, or physician referral	2012–2018	71	Caucasian (100%)	Caucasian (100%)
HDM (Spain)	Healthy male and female adult (18 years +) controls are recruited from the outpatients clinic at the Hospital del Mar in Barcelona, Spain and agree to the measurement via informed consent	Outpatient clinic Hospital del Mar Barcelona, Spain	2010–2018	81	–	Caucasian (100%)
MGH (USA)	Healthy male and female adult (18 years +) volunteers are recruited from the greater Boston, MA community and agree to the measurement via informed consent	Massachusetts General Hospital (MGH) in Boston, MA	2013–2018	81	2% Asian 7% Black 91% White	2% Asian 7% Black 91% White
GEELONG (Australia)	Healthy male (20 + years) participants were selected from within the Geelong Osteoporosis Study between 2016 and 2018 and agreed to the measurement via informed consent. An age-stratified sampling method was utilised for the broader Geelong Osteoporosis Study, involving 12 strata for each sex. Individuals were selected at random from the electoral roll	Deakin University-Barwon Health (University Hospital Geelong)	2016–2018	143	Caucasian (98%)	–

Studies included adult (18 + years) females from the USA (Minnesota) [21], healthy adult men and women (18 + years) recruited from the greater Boston community (Massachusetts) [34]; female adults (18 + years) from USA (Columbia, New York) [15]; male and female adults (18 + years) visiting outpatient clinic from Europe (Leiden University Medical Center (LUMC) [28, 35]; healthy male and female adult controls recruited from the Department of Endocrinology, Europe (Oslo) [24, 36]; healthy males and females (adult 18 + years) recruited from the outpatients clinic at the Hospital del Mar in Barcelona, Spain, and the Geelong Osteoporosis Study [37] (GOS), a population-based cohort study situated in a geographically well-defined region in Australia (Geelong).

The inclusion criterion for this study were:

1. Men or women aged 25 years and older as skeletal maturity is known to occur at 25 years of age [38].
2. Ability to ambulate independently
3. Ability to lie motionless in the supine position for 15 min
4. Measurements on the left or right tibia with OsteoProbe.

The exclusion criterion were:

1. A DXA-confirmed T-score ≤ − 2.5 at femoral neck or lumbar spine
2. Previous tibial stress fracture
3. Tibial lesion or tumour
4. Active infection, significant oedema or obesity that puts a thick layer of soft tissue over the tibial surface
5. Pregnancy
6. Secondary osteoporosis as indicated by markers for diseases:
  - Fragility fracture(s)
  - Any disorder associated with altered skeletal structure or function including the presence of chronic renal impairment (chronic kidney disease [CKD] stage IV or V), chronic liver disease, severe neuropathic disease, peripheral neuropathy, unstable cardiovascular disease, malignancy, chronic gastrointestinal disease, neoplasia, osteomalacia, hypoparathyroidism or hyperparathyroidism, acromegaly, Cushing’s syndrome, hypopituitarism, severe chronic obstruct pulmonary disease, alcoholism, or Type 1 or Type 2 diabetes, pathological fracture (e.g. due to Paget’s disease, myeloma, metastatic malignancy) or hereditary/genetic diseases that affect the skeleton
7. Undergoing treatment for blood clots or coagulation defects, or treatment with any of the following drugs:
  - Glucocorticoids (> 3 months at any time or > 10 days within the previous year)
  - Anticonvulsant therapy within the previous year
  - Supraphysiological doses of thyroid hormone causing thyroid stimulating hormone to decline below normal
  - Anabolic steroids
  - Aromatase inhibitors
  - Calcitonin
  - Calcium supplementation > 1500 mg/d within the preceding 3 months
  - Vitamin D supplementation > 2000 IU/D within in preceding 12 months
  - Bisphosphonates within previous 3 years
  - Estrogen or selective estrogen receptor modulator within the past year
  - Parathyroid hormone
  - Sodium fluoride
  - Denosumab, any use in last 12 months
  - Thiazolidinediones

Thus, data for this cross-sectional analysis of healthy participants were generated for 197 men and 282 women (ages 25 to 98 years) measured between 2011 and 2018.

## Methods

All participants were drawn from studies approved by the Ethics Committees at each institution. All participants provided written informed consent. Data were evaluated following an a posteriori method as defined by the US Clinical and Laboratory Standards Institute [39].

## Measures

### Bone Impact Microindentation (IMI)

IMI was measured using the OsteoProbe (Active Life Scientific, Inc., Santa Barbara, CA, USA). Each clinical site performed measurements using a single OsteoProbe device. The indentation site on the anterior surface of the mid-tibia was determined by measuring the midpoint from the medial border of the tibial plateau to the distal edge of the medial malleolus. Following disinfection of the area and administration of local anesthetic, the OsteoProbe was inserted through the skin and periosteum until reaching the surface of the bone at the anterior face of the mid-tibia. A minimum of eight and a maximum of 18 indentations were performed for each participant. At each of the participating research centres, a trained operator performed the measurement. A person was considered a trained operator if they had been taught to use the OsteoProbe by an Active Life qualified personnel, had measured at least 20 volunteers, and had the results of the measurement assessed and verified by Active Life, the manufacturer of the device. The recommendations for the standard procedure for using the OsteoProbe has been published elsewhere [40].

The procedure is well tolerated. An Investigational Device Exemption (IDE) clinical trial that focussed on the safety of the procedure was completed in 2020, with only one reported adverse event (classified by an independent Clinical Events Committee as “mild”), a report of joint pain with a reported pain of 1 out of 10 on the Numeric Rating Scale pain scale [41]. Other studies to report adverse events include one case of minor skin infection that was resolved with oral antibiotics, reports of minor discomfort and minor bruising that required no medical interventions [40] and an adverse event related to the local anaesthesia [34]. A prospective study of men from Australia reported that participants tolerated the procedure well, demonstrating the high feasibility of performing IMI measures [42].

### Bone Mineral Density (BMD)

Areal BMD (g/cm2) was measured at the femoral neck, total hip and lumbar spine using DXA. The DXAs at each site were: Hologic at the Mayo, Hologic at Columbia, Hologic at MGH, Hologic at LUMC, Lunar (iDEXA) at OSLO, QDR 4500 SR; and Horizon Wi, Hologic, Inc., Bedford, MA, USA at Hospital del Mar, Barcelona and Lunar (Prodigy Pro) at Geelong.

In order to compare BMD values measured using the different DXA scanners, standardised BMD (sBMD) was computed [43].

### Statistical Analysis

The original data were obtained from qualified clinical sites. A qualified clinical site is a clinical centre with one or multiple operators that had measured over 50 patients prior to collecting data for the study. BMSi data are stored locally on the medical device and is available for retrospective analysis at any time. Each qualified clinical site received the protocol and reviewed their measurement population according to the specific inclusion and exclusion criteria for the reference interval analysis for the period between and including January 1, 2010, and February 5, 2018. All data are stored in the same manner on each medical device. Each clinical site captured additional clinical and demographic variables that were stored in local institutional databases.

Each clinical site was queried to request BMSi data from the device along with associated clinical and demographic information for each de-identified subject. Each centre also provided BMD data; however; two centers measured BMD as per the National Osteoporosis Foundation guidance, “Clinician’s Guide to Prevention and Treatment of Osteoporosis”, hence only BMD values for male participants over 70 years and female participants over 65 years were available from those centres. Data at each clinical site were further evaluated retrospectively to determine which individuals met inclusion/exclusion criteria as defined below. Table 1 lists centres who qualified to participate.

The following questionnaire was used to query clinical sites for healthy reference interval participants. The following participant data were obtained from each study centre:

- Participant ID (de-identified)
- Sex (M/F)
- Age (years)
- BMD/T-Score (g/cm2/T-score) for femoral neck, total hip and lumbar spine
- Was informed consent received? (Y/N)
- Institutional Review Board approved? (Y/N) Approval ID Number (if available)
- Was the measurement made by a trained operator? (Y/N)
- Were there any device-related adverse events? (Y/N) If device-related adverse event observed, what was observed?
- Did the participant meet all of the inclusion criteria (Y/N)
- Did any of the exclusion criteria apply to the participant? (Y/N)

The original data included all individual indentations for each subject and the corresponding reference material measurements (i.e. raw data). All raw data were included in analysis (including data previously flagged by the operator as an ‘outlier’) and no raw data were excluded. All raw indentation values were re-run according to the latest version of the OsteoProbe software to recalculate the BMSi for each participant. The latest software deploys an automatic filter to identify and remove outlier indentations (the ‘filter’), thereby eliminating any potential operator inconsistencies in outlier selection.

The filter used by the OsteoProbe software to identify outliers was thoroughly reviewed by Food and Drug Administration (FDA) as part of the FDA clearance process. The filter is based on the principal that IMI indentations on bone follow a normal distribution. Furthermore, the standard deviation used by the filter is based on the upper end of the range for the observed standard deviations of OsteoProbe indentations on bone across thousands of indentations spanning animal models and human cadavers and confirmed by clinical in vivo measurements. By using the upper end of the observed standard deviations, the filter only eliminates extreme BMSi values that are highly unlikely to be true bone indentations.

Among all participants, including the sex and age-specific subgroups, BMSi values were normally distributed as indicated by Ryan-Joiner Test. BMSi scores were transformed ([z-mean]/SD) to normal score standard where z is the BMSi of a participant, and mean and SD are the average and standard deviation of the male or female cohort.

Means and standard deviations (SDs) were calculated for each of the following age groups: < 35, 35–44, 45–54, 55–64, 65–74, and ≥ 75 years. Relationships between BMSi values and age and BMD were described using Pearson’s correlation (for continuous variables) and one-way ANOVA when age was grouped into categories. Differences in BMSi between men and women were assessed using a two-sample *t*-test. A one-way ANCOVA was further conducted to compare the difference while controlling for age.

Multivariable linear regression models were developed to determine how BMSi was associated with age, sex and BMD; the residuals for the regression models were visualised for normality. Two-sided Healthy Reference Intervals for men and women were calculated whereby the lower and upper boundaries corresponding to the − 1.96 to + 1.96 SD from the mean are considered outside the 95% confidence intervals. This standard statistical method describes the distribution of BMSi in this population of healthy men and women and does not imply that individuals whose BMSi lies outside the 95% confidence interval are necessarily at risk of fracture or other adverse bone pathology.

Statistical analyses were performed using SAS, IBM SPSS Statistics (v28.0.0.0) and Minitab (v16, USA).

## Results

Descriptive characteristics for all participants are shown in Table 2.

**Table 2:** Participant characteristics (mean ± SD, [minimum, median, maximum])

	All (*n* = 479)	Men (*n* = 197)	Women (*n* = 282)
Age (yr)	56.4 ± 15.4	58.1 ± 15.0	55.3 ± 15.5
	[25.0, 59.1, 98.0]	[25.0, 61.0, 98.0]	[25.0, 58.0, 87.0]
sBMD femoral neck (g/cm2)	0.747 (± 0.306)	1.005 (± 0.187)	0.818(± 0.214)
	[0.268, 0.633, 1.500]	[0.484, 1.015, 1.400]	[0.484, 0.756, 1.500]
sBMD total hip (g/cm2)	0.975 (± 0.161)	1.063 (± 0.129)	0.914 (± 0.150)
	[0.032, 0.976, 1.389]	[0.760, 1.057, 1.389]	[0.032, 0.899, 1.281]
BMSi	81.3 (± 8.6)	84.4 (± 6.9)	79.0 (± 9.1)
	[48.1, 81.7, 101.4]	[62.3, 84.2, 101.4]	[48.1, 80.0, 101.1]

*sBMD* standardised bone mineral density; *BMSi* bone material strength index

Missing data: BMD femoral neck *n* = 45, total hip *n* = 97, lumbar spine *n* = 57

**Fig. 1:**
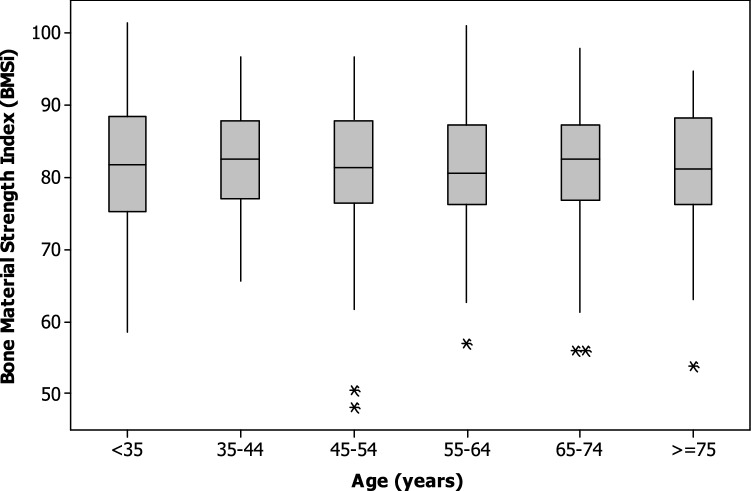
Bone material strength index (BMSi) for men and women combined, by age (decade)

**Fig. 2:**
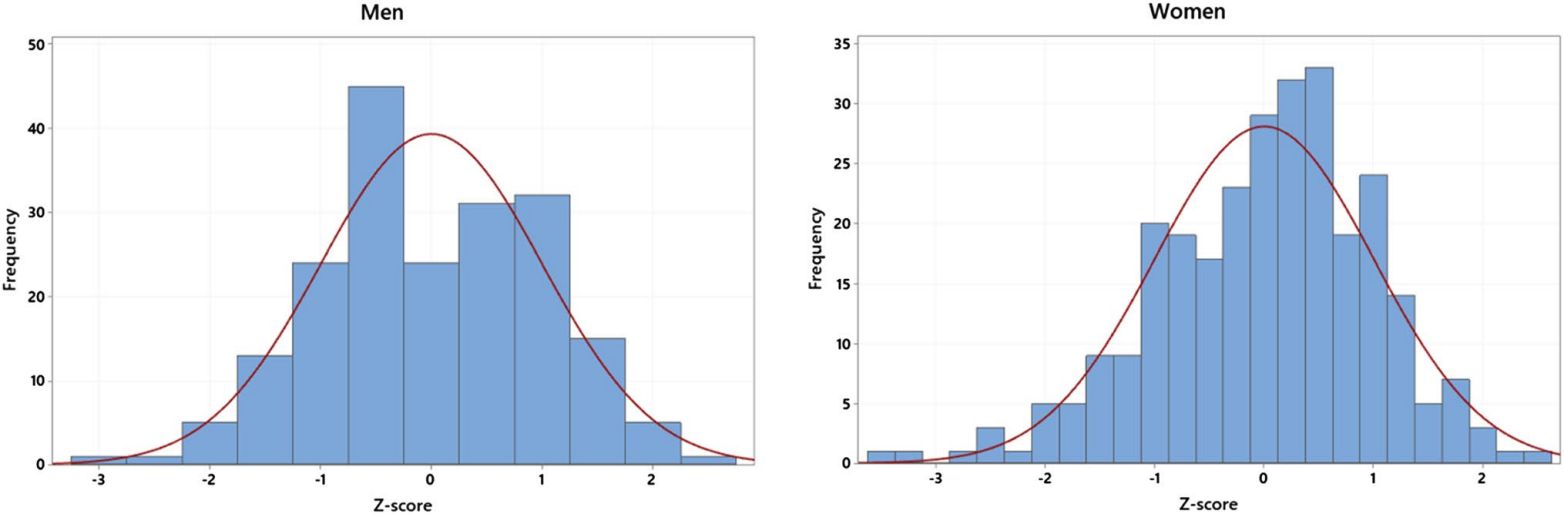
Histograms showing BMSi data for men and women

In the linear regression models, no interactions between BMSi and age or sex were identified. The correlation between BMSi and age for the whole group was not significant (*r* =  + 0.032, *p* = 0.479); the pattern was similar for each sex (men *r* =  + 0.039, *p* = 0.583; and women *r* = − 0.12, *p* = 0.841). No difference between age-categories was detected for the whole group (ANOVA *p* = 0.969) or when stratified by sex (ANOVA; men *p* = 0.862, women *p* = 0.816). Table 3 and the boxplots in Fig. 1 show no discernable inter age-group differences in BMSi. Figure 2 shows the BMSi data for men and women.

**Table 3:** Bone material strength index (BMSi) by age (decade). Data are shown for all participants and by sex

Group	Age (yr)	N	Mean	SD	Minimum	Lower quartile	Median	Upper quartile	Maximum
ALL	< 35	64	81.1	9.7	58.5	75.3	81.8	88.4	101.4
	35–44	47	81.9	8.4	65.5	77.0	82.5	87.8	96.7
	45–54	85	80.7	8.9	48.1	76.5	81.4	88.0	96.7
	55–64	114	81.1	8.4	57.0	76.3	80.5	87.3	101.1
	65–74	129	81.7	8.3	56.0	76.8	82.5	87.4	97.9
	> 75	40	81.2	8.8	53.8	76.2	81.1	88.3	94.7
MEN	< 35	23	83.6	8.4	62.3	79.0	83.2	89.2	101.4
	35–44	12	86.3	4.4	80.0	83.2	86.0	88.0	94.9
	45–54	41	83.9	6.4	72.1	79.5	82.7	89.4	96.7
	55–64	48	84.2	6.9	71.1	78.5	83.1	89.9	98.5
	65–74	53	85.0	7.05	67.2	80.2	85.6	90.5	97.9
	> 75	20	83.7	7.1	73.0	78.9	81.1	91.0	94.7
WOMEN	< 35	41	79.7	10.1	58.5	71.4	80.4	87.4	97.3
	35–44	35	80.4	8.9	65.5	71.9	80.0	87.8	96.7
	45–54	44	77.8	10.0	48.1	71.9	79.8	84.6	91.5
	55–64	66	78.8	8.7	57.0	74.0	79.3	84.0	101.1
	65–74	76	79.3	8.4	56.0	74.5	80.6	84.6	96.2
	> 75	20	77.9	9.4	53.8	73.6	79.0	85.0	93.0

In the whole group, BMSi was positively associated with BMD at the femoral neck (*r* =  + 0.223, *p* < 0.001) and at the total hip (*r* =  + 0.107, *p* = 0.037). However, when stratified by sex, correlations between BMSi and BMD at both sites were not significant; men (femoral neck; *r* =  + 0.035, *p* = 0.634, total hip; *r* =  + 0.013, *p* = 0.870), women (femoral neck; *r* = − 0.072, *p* = 0.256, total hip; *r* = − 0.090, *p* = 0.177).

Mean BMSi was greater in men than women (84.4 ± 6.9 vs 79.0 ± 9.1, *p* < 0.001). The absolute mean difference between men and women was 5.386 (*p* < 0.001)]. This significant difference persisted when adjusted for age [F (1, 476) = 49.086, *p* < 0.001].

**Fig. 3:**
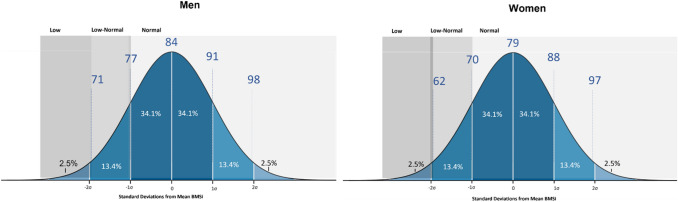
BMSi Schematic showing reference norms for men and women. *BMSi cutpoints are rounded to the nearest whole number

Calculations of Healthy Reference Intervals indicate values ranging from 71.0 to 97.9 for men and 59.8 to 95.2 for women (Fig. 3).

## Discussion

Here we present BMSi data for healthy men and women drawn from the USA, Europe and Australia. Participant ages ranged from 25 to 98 years and BMSi values ranged from 48.1 to 101.4. No associations were detected between BMSi and age within this healthy population. However, mean BMSi for women was 5.4 lower than for men. We have provided mean and SD values for each age decade, which can be used to calculate T- and Z-scores for BMSi. T-scores indicate how much a person’s BMSi varies from the mean, and a Z-score compares a person’s BMSi to the average BMSi of people of the same age. Since BMSi did not vary with age in either sex, the Z-score and T-score of BMSi are the same.

Importantly, men whose BMSi is below 71.0 and women whose BMSi is below 59.8 can be considered as having low BMSi. On the other hand, men with BMSi greater than 97.9 and women with BMSi greater than 95.2 can be considered as having high BMSi.

Aligning with our observations, no association between BMSi and age was detected in two studies limited to older women designed to investigate fractures [44] and bisphosphonate treatment [26]. It is interesting to note that while population-based data involving 252 men from Australia indicated no correlation between age and BMSi [42], when the sample size was increased to 405, a small age-related decline in BMSi of approximately 0.8 units was detected for each decade increase in age [45]. In another study, a negative association between BMSi and age (*r* =  − 0.539, *p* < 0.001) was also reported among 90 male and female patients with low bone mass [28]. Such a pattern may seem plausible, given that age-related factors such as loss of bone mass and structure [46, 47], accumulation of microcracks and deterioration of bone microarchitecture contribute to diminished bone strength [48, 49]. Notably, however, most studies reporting an association between age and BMSi have included individuals with underlying risk factors. It is plausible that an age-related decline in BMSi is more prominent in a population with comorbidities. It is also plausible that BMSi does not change with age in healthy individuals but may be altered by disease states and therefore remains a determinant of fracture risk. Further, it is likely that exclusion criteria for our study and the heterogeneity of the population might have limited the range of age-related factors that affect bone. Similarly, our exclusion criteria produced a sample where BMD at the proximal femur did not decrease with age (data not shown).

There is growing evidence that IMI may have a future in clinical practice as a complementary tool to conventional bone testing methods for predicting fracture. It has been shown to capture unique properties of bone that are not captured by DXA, particularly in populations where BMD has limited ability to discriminate fracture risk, for example, in patients with type 2 diabetes [15–19, 21] or chronic kidney disease [20, 25].

It is known that fracture risk increases with age at any given BMD [50], suggesting this increased fracture risk is a function of, at least in part, other structural or material changes not captured by DXA. This age-related increase in fracture risk is likely to be related to the cumulative effect of comorbidities on bone structure and possibly material properties as well as increased propensity to falls. BMSi is lower in the presence of certain comorbidities [16, 17, 20, 21] and, as people age and accumulate comorbidities, altered bone material properties as detected by BMSi might contribute to the observed age-related increase in fracture risk [50]. However, its diagnostic utility for fracture has not been proven, and there is not sufficient evidence to support the introduction of IMI into clinical practice**.**

This additional information provided by IMI will be useful for identifying people who would benefit from early intervention and for those at low risk, as treatment for low-risk people should be avoided. Further, the portability of the OsteoProbe device, and lack of radiation make it a practical alternative in rural settings, as access to radiation equipment and trained personnel is often inadequate.

We acknowledge several strengths and limitations in this study. The major strength of this study is that data were obtained from a heterogeneous population of participants from the USA, Europe and Australia; thus, it will be relevant for a broad population in these regions. To the best of our knowledge this study is the first to include comparable data for varying geographical regions.

We explored the associations between BMSi, age, and sex in the largest sample, and widest age range of men and women to date. Notwithstanding, the nature of this study was cross-sectional, the data were collected retrospectively, and prospective follow-up was not performed. Since it is impossible to pre-screen for fragile bone without the presence of a fracture, it is possible that some participants who met the inclusion/exclusion criteria to define healthy, and had very low BMSi, might go on to fracture. We acknowledge that it is likely that individuals with undetected bone issues may have been included in the healthy population, particularly in the female sample, where most were already visiting a hospital for some reason. Further, incomplete BMD data may have reduced the power to compare BMSi with BMD and given we did not observe an age-related decline in BMSi, the interpretation of T-scores is limited. The authors also acknowledge that the sample size is relatively small when compared to those used to develop the BMD reference data [51]. However, the sample size achieved adequate statistical power to allow meaningful conclusions. Further, IMI is a relatively new technology, currently only being used by a small number of researchers around the world, hence, there is a dearth of prospective studies, limiting their interpretation with respect to the ability of IMI to predict fractures. As more data emerge, our observations will serve as a reference study and lay the foundation for future work.

Moreover, it is not yet clear what properties of bone IMI measures. There is evidence that BMSi correlates with tissue mineral density and degree of collagen cross linking [52], cortical density [53], cortical porosity and cortical volumetric BMD [54], as detected by peripheral quantitative computed tomography. Another study assessed bone material properties in iliac bone biopsies obtained concurrently with BMSi measurements in twelve participants, showing that BMSi correlates with subperiosteal bone properties [55].

It is also possible that geographical variation in populations has a role in BMSi. In the only published study evaluating geographical variation in BMSi, significant differences in BMSi were observed between countries, with BMSi higher in healthy Spanish women than in healthy Norwegian women [44]. This suggests that the observations from any one region may not be generalisable to other populations, as there may be differences in BMSi values between geographical areas. Similarly, as participants in this study were largely Caucasian, the results may not apply more broadly to individuals of other race or ethnic origins.

In conclusion, we suggest that low BMSi corresponds to values below 71.0 for men and below 59.8 for women. This study also provides reference data that can be used to calculate T- and Z-scores for BMSi. These data will be useful for positioning individuals within the population and identify those with BMSi at the extremes of the population. Further prospective research is warranted to confirm these observations and, furthermore, extend these findings and derive optimal cut points for BMSi that discriminate fracture risk in the global population.
